# Increased expression of MMP-9 and IL-8 are correlated with poor prognosis of Bladder Cancer

**DOI:** 10.1186/1471-2490-12-18

**Published:** 2012-06-13

**Authors:** Sabrina Thalita Reis, Katia Ramos M Leite, Luís Felipe Piovesan, José Pontes-Junior, Nayara Izabel Viana, Daniel Kanda Abe, Alexandre Crippa, Caio Martins Moura, Sanarelly Pires Adonias, Miguel Srougi, Marcos Francisco Dall’Oglio

**Affiliations:** 1 Laboratory of Medical Investigation (LIM55), Urology Department, University of Sao Paulo Medical School, Sao Paulo, Brazil; 2 Uro-Oncology Group, Urology Department, University of Sao Paulo Medical School and Institute of Cancer of State of Sao Paulo (ICESP), Sao Paulo, Brazil

**Keywords:** Bladder cancer, Matrix metalloproteinase, Prognosis, Diagnosis, Gene expression

## Abstract

****Background**:**

Extracellular matrix homeostasis is strictly maintained by a coordinated balance between the expression of metalloproteinases (MMPs) and their inhibitors. The purpose of this study was to investigate whether the expression of MMP-9, MMP-2 and its specific inhibitors, are expressed in a reproducible, specific pattern and if the profiles are related to prognosis in Bladder Cancer (BC).

****Methods**:**

MMP-9, MMP-2 and its specific inhibitors expression levels were analyzed by quantitative real-time polymerase chain reaction (qRT-PCR) in fresh-frozen malignant tissue collected from 40 patients with BC submitted to transurethral resection of bladder. The control group consisted of normal bladder tissue from five patients who had undergone retropubic prostatectomy to treat benign prostatic hyperplasia.

****Results**:**

MMP-9 was overexpressed in 59.0 % of patients, and MMP-2, TIMP-1, TIMP-2, MMP-14, RECK and IL-8 was underexpressed in most of the patients. Regarding prognostic parameters we observed that high-grade tumors exhibited significantly higher levels of MMP-9 and IL-8 (p = 0.012, p = 0.003). Invasive tumors (pT1-pT2) had higher expression levels of MMP-9 than superficial tumors (pTa) (p = 0.026). The same was noted for IL-8 that was more expressed by invasive tumors (p = 0.015, p = 0.048). Most importantly tumor recurrence was related with higher levels of both MMP-9 (p = 0.003) and IL-8 (p = 0.005).

****Conclusion**:**

We have demonstrated that the overexpression of MMP-9 and higher expression of IL-8 are related to unfavorable prognostic factors of urothelial bladder cancer and tumor recurrence and may be useful in the follow up of the patients.

## **Background**

Bladder cancer (BC) is the second most common malignancy of the urinary tract. Approximately 383,300 new cases are estimated for 2011 world-wide [1]. Ninety percent of BC are urothelial carcinomas, previously named transitional cell carcinomas, and the majority are papillary low-grade, non-muscle invasive that recur in up to 80 % of cases but rarely progress to muscle invasion [2]. In contrast, 10 to 20 % of tumors are muscle invasive at diagnosis, and 50 % of patients die from metastatic disease [3]. The molecular pathways of BC have been investigated to identify new potential markers for diagnosis, disease monitoring, prognosis and development of new targeted therapies [2,3].

Degradation of basal membranes and the extracellular matrix (ECM) is essential for tumor invasion and development of metastases, and matrix metalloproteinases (MMPs) are potent proteolytic enzymes that are known to play a key role in these processes. Within the MMP family, Matrix Metalloproteinase 2 (MMP-2) (gelatinase A, 72 kDa) and Matrix Metalloproteinase 9 (MMP-9) (gelatinase B, 92 kDa) cleave type IV collagen and gelatin, which are the main structural components of the basal membrane [4]. At the post-translational level, all MMPs are under control of specific tissue inhibitors (TIMPs). TIMP-1 specifically inhibits MMP-9 and TIMP-2 inhibits MMP-2, moreover, it has been reported that Reversion-inducing cysteine-rich protein with Kazal motifs (RECK) inhibits both MMP2 and MMP-9. Considering stimulation, membrane type-1 MMP (MT1-MMP), also known as MMP14 is one of main activators, and also Interleukin-8 (IL-8) upregulates MMP2 and MMP9 in tumor cells, which is thought to be responsible for its angiogenic activity. The balance between secreted MMPs and their specific regulators plays an important role in maintaining connective tissue homeostasis in normal tissue [5].

In neoplastic diseases, an imbalance between MMPs and their regulators, leading to an excess of degradative activity, is assumed to be linked to the invasive character of tumor cells [6,7]. Expression of MMP-9 and MMP-2 has been implicated in the development and progression of many neoplasias, such as prostate [8], colorectal [9] and lung cancer [10].

The aim of the present study was to investigate whether MMP-9 e MMP-2 and its specific inhibitors, TIMP-1 and TIMP-2, MMP-14, IL-8 and RECK, are expressed in a reproducible, specific pattern in BC. Additionally, we evaluated the correlation between the expression of these genes and three important prognostic parameters, histological grade, pathological stage and angiolymphatic invasion. Also we analyzed the profile of MMPs and inhibitors with tumor recurrence.

## **Patients and Methods**

### **Patients**

The study was conducted using surgical specimens from 40 patients with BC who underwent transurethral resection in our institution between 2006 and 2008. The demographic, clinical and pathological data are described in Table 1. As control, we used normal bladder tissue from five male patients who had undergone retropubic prostatectomy to treat benign prostatic hyperplasia; the mean age of the control group was 64.0 years old, ranging from 60 to 70. These cases were randomly selected from our database. All subjects provided informed consent to participate in the study and to allow their biological samples to be genetically analyzed. Approval for the study was given by the Institutional Board of Ethics (no. 0105/09).

**Table 1:** Demographic characteristics of 40 patients with Bladder Cancer

	**Cases**
Age (years)	
Mean	69.0
Min - Max	(47–87)
Gender	
Man n (%)	31 (71.5)
Woman n (%)	9 (22.5)
Grade	
Low n (%)	18 (45.0)
High n (%)	22 (55.0)
Stage	
pTa n (%)	19 (47.5)
pT1 n (%)	13 (32.5)
pT2 n (%)	8 (20.0)
Angiolinfatic invasion	
Without n (%)	36 (90.0)
With n (%)	4 (10.0)
Recurrence	
Without n (%)	21 (67.7%)
With n (%)	10 (32.3%)

### **RNA Isolation and cDNA Synthesis**

All tumor samples were obtained from surgical specimens and immediately frozen at −170 °C in liquid nitrogen. A slide with a mirror of the frozen fragment was stained with hematoxylin and eosin to verify that the tumor represented at least 75 % of the fragment in patients with cancer and to demonstrate the absence of tumor in those without.

Total RNA was isolated with an RNAaqueous Kit (Applied Biosystems, CA, USA) according to the manufacturer’s instructions. RNA concentration was determined by 260/280 nM absorbance using a Nanodrop ND-1000 spectrophotometer (Thermo Scientific). cDNA was generated using a High Capacity cDNA Reverse Transcription Kit (Applied Biosystems, CA, USA). The reactions were incubated at 25 °C for 10 min, followed by 37 °C for 120 min and 85 °C for 5 min. The cDNA was stored at −20 °C until further use.

#### **Quantitative Real-Time PCR and Gene Expression**

Expression levels of the genes were analyzed by qRT-PCR using an ABI 7500 Fast Real-Time PCR System (Applied Biosystems). Target sequences were amplified in a 10-μl reaction containing 5 μl of TaqMan Universal PCR Master Mix, 0.5 μl of TaqMan Gene Expression Assays (primers and probes; see Table 2), 1 μl of cDNA and 3.5 μl of DNase-free water. The PCR cycling conditions were 2 minutes at 50 °C, 10 minutes at 95 °C, and then 40 cycles of 15 seconds at 95 °C and 1 min at 60 °C. A TaqMan B2M assay was utilized as the endogenous control (Table 2).

**Table 2:** Primers utilized

**Gene**	**Assay**
MMP2	Hs00234422_m1
MMP9	Hs00957562_m1
TIMP1	Hs00212624_m1
TIMP2	Hs00234278_m1
MMP14	Hs00237119_m1
RECK	Hs01019179_m1
IL8	HS99999034_m1
B2M	Hs99999907_m1

We used the ΔΔCT method to calculate the relative expression of the target genes using the formula ΔΔCT = (CT target gene, BC sample - CT endogenous control, BC sample) – (CT target gene, normal bladder tissue sample - CT endogenous control, normal bladder tissue sample sample). The fold change in gene expression was calculated as 2^-ΔΔCT^.

### **Statistical analysis**

Qualitative variables were expressed as numbers and percentages. For analysis of expression according to pathological stage, grade, angiolymphatic invasion and recurrence, we used the T test for homogeneous variables or Mann–Whitney test for heterogeneous variables. Statistical analysis was performed using SPSS 19.0 for Windows, and significance was set at p ≤0.05.

## **Results**

**Figure 1:**
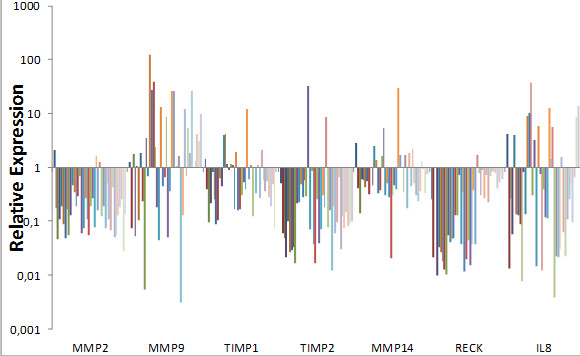
**Expression levels of the three genes in BC tissue compared with normal bladder tissue.** Fold change in expression was calculated using the 2^-ΔΔCT^ method.

MMP-9 was overexpressed in 59.0 % of patients (Figure 1). High-grade tumors exhibited significantly higher MMP-9 levels compared to low grade tumors (p = 0.012). The same was found for invasive tumors (pT1-pT2) that showed higher expression levels than superficial tumors (p = 0.026) (Table 3). Regarding tumor recurrence, we observed that patients with tumor recurrence showed higher expression of MMP-9 and IL-8 when compared with patients that not recurred (p = 0.003; p = 0.005) (Table 4). We did not find differences in gene expression related to angiolymphatic invasion.

**Table 3:** Relative expression of the genes in the malignant bladder tissue according to grade, Pathological stage and angiolinfatic invasion

**Grade Median (min-max)**				**Pathological Stage Median (min-max)**				**Angiolinfatic Invasion Median (min-max)**		
Genes	Low (n=18)	High (n=22)	p	pTa (n=19)	pT1 (n=13)	pT2 (n=8)	p	Without (n=36)	With (n=4)	p
MMP2	0.125(0.50-2.10)	0.166(0.02-1.60)	0.904	0.165(0.05-2.10)	0.109(0.02-0.34)	0.226(0.05-1.60)	0.200	0.130 (0.02-2.10)	0.229 (0.13-0.67)	0.148
MMP9	0.680(0.05-38.9)	3.291(0.03-125.1)	**0.012**	0.687(0.00-38.9)	8.532 (0.00-125)	2.938(0.18-25.8)	**0.048**	1.250 (0.00-125)	2.938 (1.01-13.2)	0.333
TIMP1	0.489(0.10-4.02)	0.508(0.07-12.01)	1.000	0.398(0.10-4.02)	0.326 (0.07-4.13)	0.967(0.16-12.0)	0.169	0.443 (0.07-12.0)	0.978 (0.44-1.91)	0.148
TIMP2	0.076(0.01-8.42)	0.110(0.01-32.5)	0.427	0.077(0.01-8.42)	0.090(0.01-0.47)	0.255(0.01-32.5)	0.247	0.085 (0.01-32.5)	0.255 (0.09-0.84)	0.101
MMP14	0.469(0.02-2.87)	0.493(0.14-30.4)	0.798	0.516(0.29-2.87)	0.336 (0.14-1.67)	0.756(0.02-30.4)	0.08	0.464 (0.02-30.4)	0.540 (0.46-1.62)	0.557
RECK	0.175(0.00-1.65)	0.129(0.01-0.88)	0.606	0.224(0.00-1.65)	0.046 (0.01-0.88)	0.240(0.01-0.71)	0.953	0.224 (0.00-1.65)	0.09(0.03-0.35)	0.772
IL8	0.075(0.00-37.2)	0.783(0.01-13.63)	**0.003**	0.087(0.00-37.2)	0.750 (0.02-13.6)	2.042(0.01-12.9)	**0.015**	0.132 (0.00-37.2)	2.412 (0.30-5.85)	0.161

Fold change in gene expression was calculated using the ΔΔCT method (QRel = 2^-ΔΔCT^).

**Table 4:** Relative expression of the genes in the malignant bladder tissue according to recurrence

	**Recurrence Median (min-max)**		
Genes	without (n=21)	with (n=10)	p
MMP2	0.106 (0.04-1.23)	0.223(0.05-0.67)	0.529
MMP9	0.976 (0.03-25.9)	6.323(0.43-125.1)	**0.003**
TIMP1	0.397 (0.08-2.06)	0.692(0.16-4.13)	0.114
TIMP2	0.075 (0.01-32.5)	0.270(0.03-0.84)	0.456
MMP14	0.516(0.02-5.31)	0.419(0.28-1.62)	0.390
RECK	0.288(0.01-1.65)	0.090(0.01-0.81)	0.681
IL8	0.096(0.00-13.6)	0.783(0.11-37.2)	**0.005**

Fold change in gene expression was calculated using the ΔΔCT method (QRel = 2^-ΔΔCT^).

IL-8 was underexpressed in the majority of cases (67.5 %) compared with normal controls (figure 1). We also found correlation of IL-8 expression in relation to tumor grade. High grade tumors showed significantly higher levels of IL-8 (p = 0.003). The same was detected for pT1 and pT2 invasive tumors that showed higher expression of IL-8 when compared to noninvasive tumors pTa (p = 0.015, p = 0.048). Regarding tumor recurrence, patients who have recurred showed higher expression of IL-8 (p = 0.005). Again, there was no correlation between IL-8 expression and angiolymphatic invasion (p = 0.161) (Table 3).

Almost all patients showed underexpression of MMP-2, TIMP-1, TIMP-2, MMP-14 and RECK (92,5 %, 72.5 %, 95.0 %, 72.5 % and 97.4 %) (Figure 1). We did not find significant differences between the expression of these genes with histological grade, pathological stage, angiolymphatic invasion (Table 3), or tumor recurrence (Table 4).

## **Discussion**

The present study demonstrates that MMP-9 is overexpressed and TIMP-1 and RECK are underexpressed in malignant bladder tissues compared with normal bladder tissues. We demonstrated also that MMP-2 and its regulators (TIMP-2, MMP-14 and IL-8) are underexpressed in BC. Most importantly we found increased levels of MMP-9 and IL-8 in high grade, invasive and recurrent tumors.

Extensive studies have revealed that malignant invasion and metastasis require ECM degradation, mainly by MMPs [11]. Excessive or inappropriate expression of MMPs may contribute to the pathogenesis of cancer in a wide variety of cases by facilitating tissue degradation. Currently, there are more than 20 identified MMPs that can be categorized by substrate specificity. Despite the clinical significance of the pathogenetic impact of MMPs in human cancer, including BC, only a few studies of MMPs are available in the literature, and those mainly analyze protein expression [12].

In the present study, we have shown not only that MMP-9 is overexpressed in cancer tissue, but also that MMP-9 levels are significantly higher in patients with high grade and infiltrative tumors, two very important prognostics factors.

The same occurred considering IL-8 expression, besides the fact that IL-8 is underexpressed in cancer tissue, the relation between higher IL-8 expression and unfavorable prognostic characteristics could be related to the fact that IL-8 is important for tumor progression, but not for tumor initiation or promotion.

MMP-9 and IL-8 expression levels increased together with tumor invasiveness, suggesting that they are probably associated with a worse outcome in BC. This is the first time that a study has shown a correlation between high grade, invasive tumors and IL-8 mRNA expression in BC. Increased MMP-9 expression has also been shown to have an independent prognostic impact on operable non-small cell lung cancer [13] and renal cell carcinoma [14], and increased IL-8 expression has been associated with worse prognostic of colon cancer [15].

Another important finding of our study was the relationship between MMP-9 and IL-8 higher levels and tumor recurrence during a long follow-up period (mean of 40 months). The mean expression of MMP-9 and IL-8 in patients with recurrence was greater than six times that observed in patients without recurrence. This result might reflect an important biological phenomenon that could be confirmed in larger studies and that could become a tool to identify patients likely to progress. MMPs are abundantly expressed in malignant tumors regardless of their origin, and a significant correlation between increased MMP expression and a poor prognosis in terms of survival has been demonstrated in several cases [16,17]. As a result, the possibility of MMPs being used as tumor markers has been suggested.

At the post-translational level, all MMPs are under the control of specific tissue inhibitors (TIMPs) that bind proximal to the catalytic domain of MMPs, preventing substrate attachment. TIMPs are not simply regulators of MMP activity; they also have multifunctional roles that include cell growth promotion and inhibition of angiogenesis [18]. Four TIMPs have been identified. They inhibit all MMPs, forming non-covalent complexes with the active forms. Among them, TIMP-1 selectively binds pro-MMP-9 and is considered the main inhibitor of MMP-9, TIMP-2 binds pro-MMP-2. The reversion-inducing cysteine-rich protein with Kazal motifs (RECK) gene was identified as an inducer of a flat morphology in v-Ki ras–transformed NIH3T3 cells [19]. RECK expression is observed in normal human organs; however, several oncogenic factors, such as activated Ras [20], EBV latent membrane protein 1 [21], and HER-2/neu [22] suppress the expression of RECK. Additionally, it has been reported that RECK overexpression decreases the amount of active MMP-2 and MMP-9 in conditioned medium and inhibits metastatic activity *in vitro*[23] and *in vivo*[24]. Considering activation of MMPs, MT1-MMP is a key enzyme in tumor angiogenesis and metastasis: it hydrolyzes a variety of ECM components, and is a physiological activator of pro-MMP-2 and MMP-9. IL-8 is involved in the transcription induction of MMP-2 increasing stromal invasion by tumor cells facilitating angiogenesis and development of metastasis.

Interestingly, our results demonstrate that there is an upregulation of MMP-9 and a downregulation of its specific inhibitors, TIMP-1 and RECK, in BC. Otherwise, we demonstrated that the low expression of TIMP-2, MMP-14 and IL-8 may be responsible for the decreased MMP-2 expression in BC tissue.

## Conclusions

In conclusion, this is the first study to indicate the prognostic relevance of MMP-9 and IL-8 mRNA expression in BC, as demonstrated by the significant correlation between its expression and invasive, high grade tumors and also BC recurrence. These findings suggest the possibility of using the profiles of MMP-9 and IL-8 as prognostic marker and, in the future, might inspire the development of targeted drugs.

## **Abbreviations**

BPH: Benign prostatic hyperplasia; cDNA: Complementary deoxyribonucleic acid; ECM: Extracellular matrix; MMP: Matrix metalloproteinase; BC: Bladder Cancer; qRT-PCR: Quantitative real-time polymerase chain reaction; RECK: Reversion-inducing cysteine-rich protein with Kazal motif; RNA: Ribonucleic acid; TIMP-1: Tissue inhibitor of metalloproteinases; TIMP-2: Tissue inhibitor of metalloproteinases; IL-8: Interleukin 8.

## **Competing interests**

The authors declare that they have no competing interests

## **Authors' contributions**

AC, JPJr, DKA-conception and design: -acquisition of patients and data. STR, KRL-Drafting of the manuscript. STR, NIV, CMM-molecular genetic studies. SPA- Administration support. STR- Statistical Analysis. MFD, AC, KRL-critical revision and important intellectual content MS-supervision. All authors read and approved the final manuscript.

## Pre-publication history

The pre-publication history for this paper can be accessed here:

http://www.biomedcentral.com/1471-2490/12/18/prepub
